# Anti-adipogenic Effects and Mechanisms of Ginsenoside Rg3 in Pre-adipocytes and Obese Mice

**DOI:** 10.3389/fphar.2017.00113

**Published:** 2017-03-08

**Authors:** Longyun Zhang, Lijuan Zhang, Xiaoyong Wang, Hongwei Si

**Affiliations:** Department of Family and Consumer Sciences, Tennessee State University,Nashville, TN, USA

**Keywords:** ginsenoside Rg3, anti-adipogenic, PPAR gamma, pre-adipocytes, obese mice

## Abstract

Red or black ginseng has been reported more powerful than white/fresh ginseng in dealing with various diseases/conditions including obesity. The major reason is that heating/steaming, the process of making red or black ginseng, produces large amount of bioactive compounds including ginsenoside Rg3 (Rg3), which are trace in fresh or white ginseng. In the present study, Rg3 was applied both in pre-adipocytes and obese mice to investigate the anti-adipogenic effects and relevant mechanisms. Our results show that Rg3 dose-dependently inhibited cell differentiation both in 3T3-L1 cells (30, 50, and 100 μM) and human primary pre-adipocytes (10, 20, and 30 μM). This inhibitory effect is accompanied by the attenuation of the expressions of adipogenic markers including peroxisome proliferator-activated receptor gamma (PPAR-γ), CCAAT/enhancer binding protein alpha (C/EBP-α), fatty acid synthase (FAS), fatty acid binding protein 4 (FABP4) and perilipin. Although dietary intake of Rg3 (0.1 mg Rg3/kg diet, 8 weeks) did not significantly affect body weight gain, fat pads and food intake as well as of PPAR-γ expression in fat tissues, we found that hepatic PPAR-γ and C/EBP-α protein expressions and hepatic glutathione reductase and glutathione *S*-transferase, two major antioxidants molecules were significantly reduced by Rg3. These results suggest that ginsenoside Rg3 may be a potential agent in reducing/preventing obesity.

## Introduction

Adipogenesis, the process of adipocytes differentiated from pre-adipocytes, plays a key role in the adult obesity development because obese subjects have more fat cells compared to the lean subjects (van Harmelen et al., 2003). Moreover, approximately 10% of fat cells turnover annually in both lean and obesity adults, but obese adults recruited more adipocytes than lean adult (Spalding et al., 2008). This is further supported by that adipocyte number is increased in response to a high-fat diet in adult rats (Faust et al., 1978). The adipogenesis and lipogenesis (the process of fatty acid, triglyceride synthesis, and fat drop packaging) are regulated by transcriptional cascades PPAR-γ and CCAAT/enhancer binding proteins (C/EBPs), which are accompanied by a dramatic changes of expressions of FAS, FABP4, perilipin, adiponectin, and perilipin (Gregoire et al., 1998). Adipogenic induction rapidly induces expressions of C/EBP-β and C/EBP-δ at early clonal expansion and growth arrest. Increased C/EBP-β and C/EBP-δ target downstream key adipogenic transcriptional regulators C/EBP-α, PPAR-γ and the regulator of lipogenic genes sterol-regulatory-element-binding protein 1. PPAR-γ activates the promoter of the genes encoding C/EBP-α and vice versa, creating a positive-feedback loop. Increased C/EBP-α and PPAR-γ induce the expression of genes that are involved in insulin sensitivity, lipogenesis, and lipolysis, including those encoding glucose transporter, FABP4, lipoprotein lipase, perilipin and the secreted factors adiponectin and leptin (Lowe et al., 2011).

Ginseng has long history dealing with human health, and various health benefits of ginseng have been reported on cardiovascular disease (CVD) (Kim, 2012; Lee and Kim, 2014), type 2 diabetes (T2D) (Jeon et al., 2013), immune function (Lui et al., 2013; Jia et al., 2014), erectile dysfunction (Low and Tan, 2007; Jang et al., 2011; Choi et al., 2013), neural function (Liao et al., 2002; Zheng et al., 2011; Ong et al., 2015), and obesity (Yun et al., 2004; Kim et al., 2009). Although ginseng contains varieties of bioactive compounds such as ginseng saponins, peptides, polysaccharide, fatty acids, vitamins, alkaloids, lignans, and flavonoids (Wang et al., 2012), Saponins, are named as ginsenosides in 1957 by Brekhamn, are the major contributors of the ginseng beneficial effects (Shibata et al., 1963; Cho et al., 2013). Over 100 ginsenosides have been identified since the first description in the 1960s (Shin et al., 2015). Ginsenoside Rg3 (Rg3) is trace naturally occurring in fresh and white ginseng, but majorly comes from the heat processing of protopanaxadiol (PPD) ginsenosides, such as Rb1, Rb2, Rc, and Rd from red ginseng. Rg3 has been reported in inhibiting breast cancer cell proliferation (Wang et al., 2008) and Alzheimer’s amyloid beta peptide in mice (Chen et al., 2006) as well as vascular inflammation in cells and animals (Cho et al., 2014). While only one study reported the anti-obesity effect of Rg3 in 3T3-L1 cells (Hwang et al., 2009), studies in primary human cells, animals, and humans are still lacking. In the present study, Rg3 were treated in 3T3-L1 cells, human primary pre-adipocytes (HPAs), and obese mice. We found that Rg3 dose-dependently inhibited cell differentiation both in 3T3-L1 cells and HPAs, which was accompanied by the reductions of proteins and mRNA expressions of PPAR-γ, C/EBP-α, FAS, and FABP4. Rg3 also reduced hepatic PPAR-γ and C/EBP-α protein expressions in obese mice. The changes of these key molecules of adipogenesis provide critical clues for us to understand how Rg3 inhibits fat development in molecular level.

## Materials and Methods

### Reagents

Dulbecco’s modified Eagle’s medium from Gibco (Grand Island, NY, USA) was used for culture. Fetal Bovine Serum from Mediatech (Manassas, VA, USA) and penicillin streptomycin from Gibco (Carlsbad, CA, USA) were used for enhancing and maintaining the cell culture. Pierce BCA protein assay kit and SuperSignal West Dura Extended Duration Substrate kit were purchased from ThermoFisher Scientific (Rockford, IL, USA). Mammalian protein extraction buffer were purchased from GE Healthcare Bio-Sciences (Piscataway, NJ, USA). Specific antibodies that recognized the PPAR-γ, C/EBP-α, FAS, FABP4, Perilipin, β-actin were purchased from Cell Signaling Technology (Danvers, MA, USA). Insulin from Roche (Mannheim, Germany), and IBMX, dexamethasone, and rosiglitazone from Sigma (St Louis, MO, USA) were used to induce adipocyte differentiation. Ten percent buffered formalin phosphate and 2-propanol were purchased from Fisher Scientific (Fair Lawn, NJ, USA). Oil-red O solution was purchased from Electron Microscopy Science (Hatfield, PA, USA). TACS MTT cell proliferation assay kit was purchased from Trevigen (Gaithersburg, MD, USA). Dimethyl sulfoxide (DMSO) was purchased from Sigma (St Louis, MO, USA). RNeasy Mini Kit was purchased from Qiagen (Valencia, CA, USA) and iTaq Universal SYBR Green One-Step Kit was purchased from Bio-Rad (Hercules, CA, USA). Ginsenoside Rg3 was purchased from ChromaDex (Irvine, CA, USA) and was dissolved in DMSO, aliquots at 100 mM was storage in −20°C freezer.

### 3T3-L1 Cell Culture

3T3-L1 cells were purchased from the American Type Culture Collection (ATCC, Manassas, VA, USA). Passages between 5 and 25 were used in all experiments. The cells were cultured in DMEM containing 10% fatal bovine serum and 1% penicillin streptomycin mixture in an incubator with 5% CO_2_ at 37°C.

### Human Primary Pre-adipocytes Cell Culture

Human primary pre-adipocytes were purchased from ATCC (Manassas, VA, USA). Cells were maintained at 37°C and 5% CO_2_ in Fibroblast Basal Medium plus Fibroblast Growth Kit–Serum-Free (ATCC, KS-201-040, purchased from ATCC) with biotin (33 μM) and pantothenate (17 μM). Passages between 3 and 6 were used in all experiments.

### Pre-adipocyte Differentiation

3T3-L1 or HPAs were plated in 12-well plates, and adipocytes differentiation was induced with a hormone cocktail containing 0.5 mM IBMX, 1 μM dexamethasone, 1 μM rosiglitazone, and 10 μg/mL insulin (MDI, at day 0). After 3 days, the medium was changed to normal medium containing 10 μg/mL insulin. 3T3-L1 cells need 7 days and HPAs need 10 days to accomplish the differentiation process. Both cells were treated with indicated stimuli during the whole differentiation process from Days 0 to 7 or Day 10 as previously described (Zebisch et al., 2012).

### MTT Assay

Cells were cultured in 12-well plates with different dosages of Rg3 from 30 to 300 μM. Treated cells were incubated with 100 μl MTT reagent per well for 4 h till purple dye was visible. Five hundred microliter of detergent reagent was added into each well and incubated in the dark for 4 h. The relative cell viability was determined by the absorbance at 570 nm using Synergy H1 hybrid reader (BioTek Instruments, Inc., Winooski, VT, USA).

### Oil-Red O Staining

After cells accomplished the differentiation process, cells were fixed with 10% buffered formalin phosphate for 1 h at 4°C. The cells were stained with Oil-red O dye. Fat droplets in the adipocytes were stained with red color. The dye was then dissolved in 100% isopropanol and relative fat accumulation was measured by absorbance at 490 nm using Synergy H1 hybrid reader (BioTek Instruments, Inc., Winooski, VT, USA).

### Animal Feeding and Tissue Collection

Four week-old male obese leptin-deficient mice (B6.Cgx-*Lep*^ob^/J, ob/ob), Jackson Laboratory (Bar Harbor, ME, USA) were feed either with AIN-93G mineral mix standard food (0.88 kcal/g), or standard food containing 0.01% ginsenoside Rg3 (0.1 mg Rg3/kg diet, w/w) for 8 weeks. Body weight and food intake were recorded weekly throughout the study. After 8-week treatment, mice were euthanized with CO_2_. All experimental procedures were approved by the Institutional Animal Care and Use Committee at Tennessee State University in accordance with the National Institutes of Health Guidelines for the Care and Use of Laboratory Animals. Blood, fat tissue, liver was harvested and prepared for blood glucose, antioxidant activity, and relevant proteins analysis.

### Protein Extraction and Western Blotting

Treated cells were washed once with PBS, and scraped into mammalian protein extraction buffer. Cells were homogenized by sonication on ice and centrifuged 10 min at 12,000 × *g* at 4°C. Animal tissue was mixed with mammalian protein extraction buffer and homogenized on ice. The supernatant was collected as protein sample after centrifuging 10 min at 12,000 × *g*. Protein concentration was measured by Pierce BCA protein assay kit. Protein sample was mixed with Laemmli sample buffer and heated at 95°C for 5 min, and subjected to western blot analysis. Samples were separated by 10% SDS-PAGE and the membrane was blocked for 1 h with PBS (TBST) containing 5% skim milk at room temperature. After washing three times with TBST, the membrane was incubated with relevant primary antibody at 4°C for overnight. The membrane was washed three times with TBST and then incubated with secondary antibody for 1 h at room temperature. Specific bands were detected by SuperSignal West Dura chemiluminescence (ThermoFisher Scientific, USA) and visualization was performed by exposure of the membranes to X-ray films. The same set of samples to detect different proteins. The same membrane was stripped using a stripping buffer for 30 min, and blocked and probed with a new primary antibody to detect the next specific protein. Band intensities were quantified by ImageJ software (National health institute, USA) (Lee et al., 2013).

### Quantitative Real Time PCR

Treated cells were used to isolate total RNA using RNeasy Mini Kit instructed by the manufacturer’s protocol. Thirty nanograms RNA temples were used for each treatment. The reaction and cycling conditions for one-step real time PCR are: cDNA synthesis 10 min at 50°C; reverse transcriptase inactivation 5 min at 95°C; 45 cycles for PCR and detection, 10 s at 95°C, 30 s at 60°C. Designed primers PPAR-γ forward: TCG CTG ATG CAC TGC CTA TG, PPAR-γ reverse: GAG AGG TCC ACA GAG CTG ATT (Chen et al., 2013); β-actin forward: AGC CTT CCT TCT TGG GTA TGG, β-actin reverse: CAC TTG CGG TGC ACG ATG GAG (Bae C.R. et al., 2014) were used for the real time PCR by using iTaq Universal SYBR Green One-Step Kit. Relative gene expression was normalized by the expression of β-actin, and was calculated by using 2^-ΔΔCt^ method.

### Statistical Analysis

Data are presented as mean ± SEM of at least three independent experiments performed in triplicate. Values of *p* < 0.05 were considered to be significant.

## Results

### Ginsenoside Rg3 Dose-Dependently Inhibits Cell Differentiation in 3T3-L1 Cells

**FIGURE 1:**
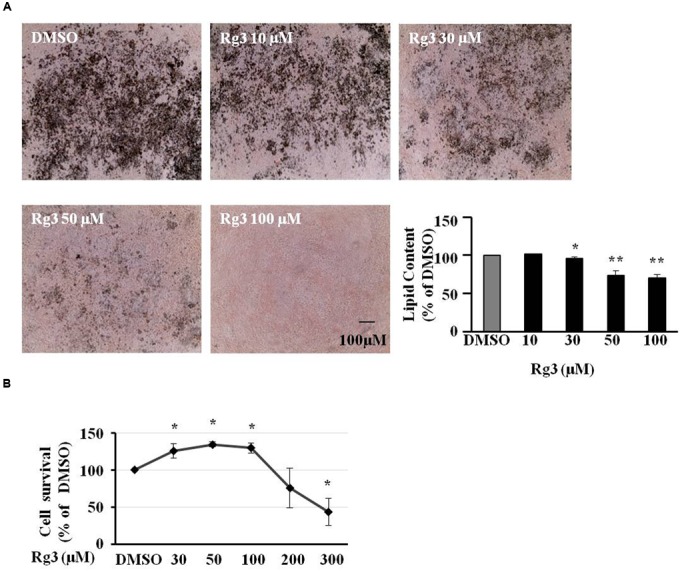
**Ginsenoside Rg3 dose-dependently inhibits cell differentiation in 3T3-L1 cells.** The cells stained with Oil-red O were dissolved in isopropanol and relative fat accumulation was measured by absorbance using Synergy H1 hybrid reader at 490 nm. Oil-Red O representative images of lipid accumulation and the average bar graph were shown (**A**, 40× magnification). Cell toxicity study was measure by a MTT assay **(B)**. Changes by treatment were expressed as the percentage of dimethyl sulfoxide (DMSO) control. Data are expressed as mean ± SE, *n* = 4. ^∗^*P* < 0.05, ^∗∗^*P* < 0.01 vs. DMSO control.

Ginsenoside Rg3 at 10, 30, 50 or 100 μM was added in the medium from day 0 (adding differential inducer MDI mixture) in 3T3-L1 cells. Various treatments were added to new medium on days 3, 5, and 7 to replace medium in cells. On day 10, cells were stained by Oil-red O to take images and measure fat accumulation. Accumulated fat droplets were dissolved in 100% isopropanol, and the intensity was measured using a Synergy H1 microplate reader. As shown in **Figure 1A**, Rg3 dose-dependently inhibited MDI-induced intracellular lipid droplets, particularly at 100 μM, Rg3 almost abolished the MDI effect. To make sure the anti-adipogenic effect of Rg3 is not because of killing cells, we conducted a MTT cell viability assay. We found that all concentrations (10–100 μM) of Rg3 having anti-adipogenic effect slightly increased cell proliferation (**Figure 1B**). These results indicated that Rg3 prevented pre-adipocytes differentiation in 3T3-L1 cells in a dose-dependent manner.

### Ginsenoside Rg3 Dose-Dependently Suppresses Protein Expressions of PPAR-γ and C/EBP-α in 3T3-L1 Cells

**FIGURE 2:**
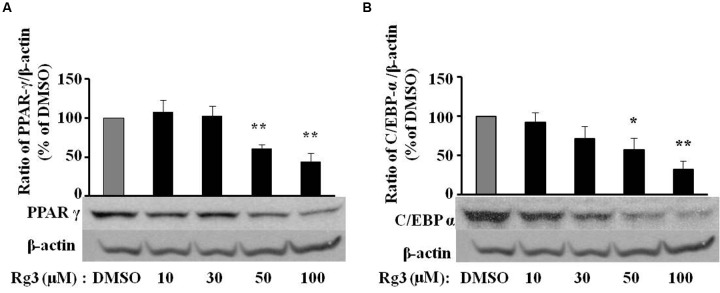
**Ginsenoside Rg3 dose-dependently suppresses protein expressions of PPAR-γ (A)** and C/EBP-α **(B)** in 3T3-L1 cells. On day 10, cells treated with various concentrations of Rg3 were collected to measure PPAR-γ and C/EBP-α protein expressions by western blotting and normalized by β-actin expression. The same membrane was stripped using a stripping buffer and blocked and probed with a new primary antibody to detect the next specific protein. Changes by treatment were expressed as the percentage of DMSO control. Data are expressed as mean ± SE, *n* = 3. A set of representative images and bar graph were shown. ^∗^*P* < 0.05, ^∗∗^*P* < 0.01 vs. DMSO control.

Because of the key roles of PPAR-γ and C/EBP-α in adipogenesis, we checked whether the Rg3 anti-adipogenic effect was companied by change of PPAR-γ and C/EBP-α protein expressions by Western blot. We found that MDI-induced PPAR-γ (**Figure 2A**) and C/EBP-α (**Figure 2B**) protein expressions were dose-dependently reduced by Rg3 in 3T3-L1 cells, a very similar pattern of the inhibitory effect of Rg3 in fat accumulation. PPAR-γ (**Figure 2A**) and C/EBP-α (**Figure 2B**) protein expression was significantly reduced to 61% (*p* < 0.05) and 57% (*p* < 0.05) of DMSO control, respectively, by Rg3 at 50 μM.

### Ginsenoside Rg3 Reduces PPAR-γ mRNA Expression in 3T3-L1 Cells

**FIGURE 3:**
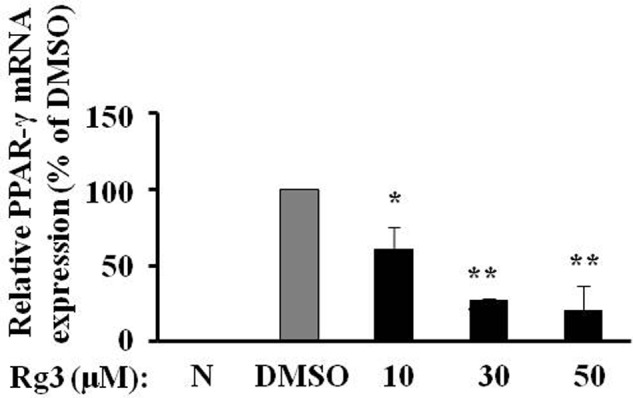
**Ginsenoside Rg3 reduces PPAR-γ mRNA expression in 3T3-L1 cells.** On day 10, cells treated with various concentrations of Rg3 were collected to measure PPAR-γ mRNA expression by quantitative real time PCR and normalized by β-actin expression. The same membrane was stripped using a stripping buffer and blocked and probed with a new primary antibody to detect the next specific protein. Changes by treatment were expressed as the percentage of DMSO control. Data are expressed as mean ± SE, *n* = 3. ^∗^*P* < 0.05, ^∗∗^*P* < 0.01 vs. DMSO control.

To investigate whether Rg3 inhibits PPAR-γ protein level via a transcriptional mechanism, we measured PPAR-γ mRNA expression in 3T3-L1 cells using quantitative real time PCR. Exposure of 3T3-L1 cells to various concentrations of Rg3 for 10 days, the same duration used to study the fat accumulation and PPAR-γ protein expression, Rg3 dose-dependently inhibited MDI-increased PPAR-γ mRNA expression, particularly reduced to 20% of MDSO control at 50 μM (**Figure 3**). This is very consistent with its effects on fat accumulation and PPAR-γ protein expression, suggesting that Rg3 inhibits PPAR-γ expression at the transcriptional level.

### Ginsenoside Rg3 Dose-Dependently Attenuates Protein Expressions of Fat Packing Proteins in 3T3-L1 Cells

**FIGURE 4:**
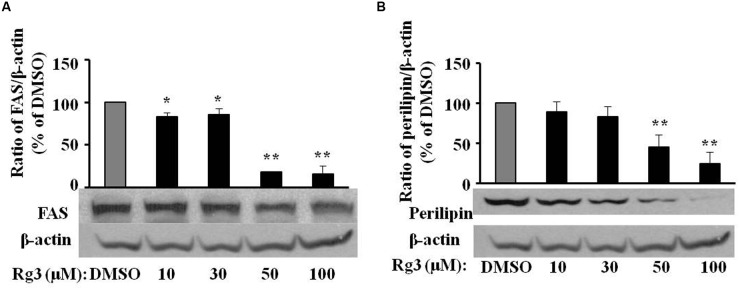
**Ginsenoside Rg3 dose-dependently attenuates protein expressions of fat packing proteins FAS (A)** and perilipin **(B)** in 3T3-L1 cells. On day 10, cells treated with various concentrations of Rg3 were collected to measure FAS and perilipin protein expressions by western blotting and normalized by β-actin expression. The same membrane was stripped using a stripping buffer and blocked and probed with a new primary antibody to detect the next specific protein. Changes by treatments were expressed as the percentage of DMSO control. Data are expressed as mean ± SE, *n* = 3. A set of representative images and bar graph were shown. ^∗^*P* < 0.05, ^∗∗^*P* < 0.01 vs. DMSO control.

Fatty acid synthase (An enzyme to catalyze the synthesis of palmitate from acetyl-CoA and malonyl-CoA), FABP4 (A carrier protein for fatty acids), and perilipin (a protein that coats lipid droplets in adipocytes) are major makers of lipogenesis and fat drop packing. We measured the protein expressions of these proteins in 3T3-L1cells treated by various levels of Rg3. Rg3 dose-dependently inhibited the protein expressions of FAS (**Figure 4A**) and perilipin (**Figure 4B**), the same pattern of Rg3 on fat accumulation, PPAR-γ and C/EBP-α expressions in 3T3-L1 cells.

### Ginsenoside Rg3 Dose-Dependently Reverses Lipid Accumulation, Protein Expressions of Differentiation Markers in HPAs

**FIGURE 5:**
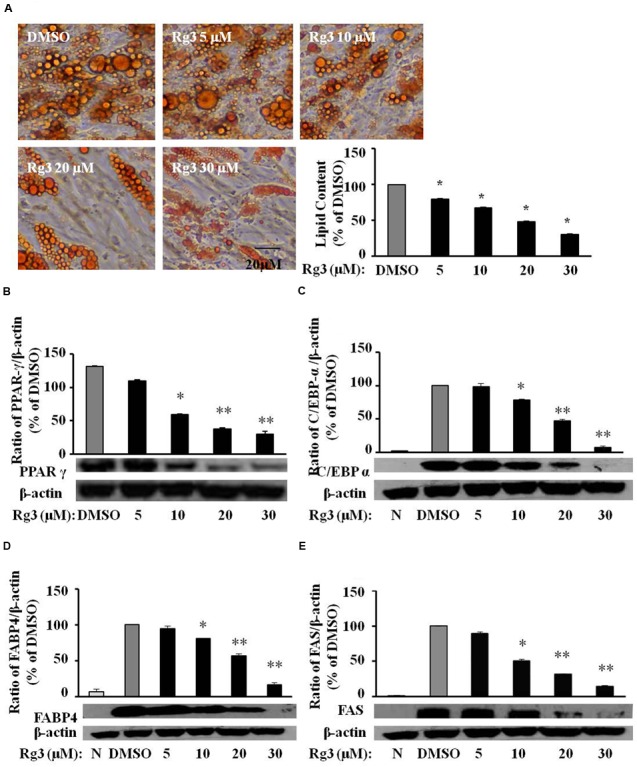
**Ginsenoside Rg3 dose-dependently reverses lipid accumulation, protein expressions of differentiation markers in HPAs.** The cells stained with Oil-red O on day 15 were dissolved in isopropanol and fat accumulation was measured by absorbance using Synergy H1 hybrid reader at 490 nm. Oil-Red O representative images of lipid accumulation and the average bar graph were shown (**A**, 200× magnification); Protein expressions of PPAR-γ **(B)**, C/EBP-α **(C)**, FABP4 **(D)**, and FAS **(E)** were determined by western blotting and normalized to β-actin expression. The same membrane was stripped using a stripping buffer and blocked and probed with a new primary antibody to detect the next specific protein. Changes by treatment were expressed as the percentage of DMSO control. Data are expressed as mean ± SE, *n* = 3. A set of representative images and bar graph were shown. ^∗^*P* < 0.05, ^∗∗^*P* < 0.01 vs. DMSO control.

3T3-L1 cells are rodent cell lines, and concentrations of anti-adipogenic effects of Rg3 are high (30–100 μM), which are not realistic to get through dietary intake of ginseng or ginsenoside Rg3. We would like to know whether HPAs are more sensitive than 3T3-L1 cells in the anti-adipogenic effect of Rg3. Indeed, comparing to the required Rg3 concentrations (30–100 μM) in 3T3-L1 cells, Rg3 inhibited differentiation at much lower concentrations (10–30 μM) in HPAs (**Figure 5A**). Moreover, Rg3 dose-dependently reduced DMI-increased proteins expressions of PPAR-γ (**Figure 5B**), C/EBP-α (**Figure 5C**), FABP4 (**Figure 5D**), and FAS (**Figure 5E**), the same pattern of Rg3 on fat accumulation in HPAs. These results suggest that Rg3 exerts anti-adipogenic effect in HPAs with similar mechanisms in 3T3-L1 cells, but needs much lower levels.

### *In vivo* Effects of Ginsenoside Rg3

**FIGURE 6:**
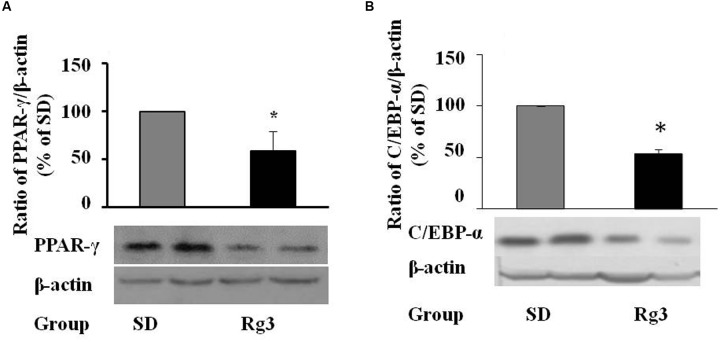
***In vivo* effects of dietary ginsenoside Rg3 intake.** After 8-week dietary administration of Rg3 (0.1 mg/kg diet), the liver PPAR-γ **(A)** and C/EBP-α **(B)** protein expression in mice was determined by western blotting and normalized to β-actin expression. The same membrane was stripped using a stripping buffer and blocked and probed with a new primary antibody to detect the next specific protein. Body weight gain, food intake, fasting blood glucose, and hepatic antioxidant were measured and are shown in **Table 1**. Data are expressed as mean ± SE, *n* = 12. A set of representative images and bar graph were shown. ^∗^*P* < 0.05, ^∗∗^*P* < 0.01 vs. standard diet (SD) control.

To confirm the anti-adipogenic effect of Rg3 *in vivo*, we tested whether dietary supplementation of Rg3 inhibits PPAR-γ and C/EBP-α expressions and thereby prevents obesity in ob/ob obese mice, a widely used obese animal model. We found that dietary intake of Rg3 (0.1 mg Rg3/kg diet, 8 weeks) significantly reduced PPAR-γ (**Figure 6A**) and C/EBP-α (**Figure 6B**) protein expression in liver, the major organ of adipogenesis, although we did not found significant change of PPAR-γ expression in fat tissues. As shown in **Table 1**, we did not observe significant changes in body weight and food intake by Rg3, however, the fasting glucose was reduced by Rg3 (*P* = 0.091) at the end of the experiment. In addition, we found that hepatic GR and GST, two major antioxidants molecules, were significant decreased in Rg3 group. These results are similar with previous study that the obese-increased hepatic GR and GST were reversed by dietary intake of grape seed extract in obese rats (Fernandez-Iglesias et al., 2014).

**Table 1:** Measurements of mice.

Group	Body weight gain (g/mouse)	Food intake (g/mouse/day)	Fasting blood glucose (mg/dL)	Liver glutathione reductase (nmol/min/mg protein)	Liver glutathione *S*-transferase (nmol/min/mg protein)
SD	21.58 ± 1.85	3.85 ± 0.30	160.13 ± 16.94	50.22 ± 3.77	1171.84 ± 53.31
Rg3	23.53 ± 1.65	3.87 ± 0.27	142.42 ± 11.16	30.99 ± 1.42^∗∗^	800.20 ± 17.22^∗∗^

*^∗∗^P < 0.01.*

## Discussion

In the present study, we reported for the first time that ginsenoside Rg3 inhibited pre-adipocyte differentiation in HPAs at much lower concentrations compared to the concentrations worked in rodent pre-adipocytes 3T3-L1 cells. We are also the first reporter that dietary supplementation Rg3 attenuated hepatic protein expression of PPAR-γ, a key regulator of adipogenesis and fat metabolism, as well as the improvements in fasting blood glucose and hepatic antioxidants GR and GST in obese mice. We also found that Rg3 dose-dependently inhibited fat accumulation, expressions of PPAR-γ, C/EBP-α, FAS, and perilipin in 3T3-L1 cells. These results suggest that ginsenoside Rg3 may be a potential agent in reducing/preventing obesity.

Ginsenoside Rg3 naturally occurs at very low level in American ginseng root extract (0.06%) (Wang et al., 2008) and berry (0.03 mg/g) (Xie et al., 2007), but not detectable (Kim et al., 2013) or trace (Kim et al., 2016) in Asian ginseng. However, heating/steaming, the traditional method in processing red ginseng (steaming fresh ginseng at 95–100°C for a reasonable time) and black ginseng (nine-time repetitive steaming white ginseng at 95–100°C for 3 h) (Gui and Ryu, 2014), dramatically increased Rg3 content both in Asian ginseng and American ginseng. For instance, 2 h steaming increased Rg3 from 0.06 to 5.9% in American root (Wang et al., 2008). While Rg3 is low in white Asian ginseng (0.004%), Rg3 (20S), and Rg3 (20R) can be 2.797 and 0.610%, respectively, in red Asian ginseng (Kim et al., 2016). Moreover, Rg3 level in black Asian ginseng can be 3–10 times of the level in red Asian ginseng (Jo et al., 2012; Lee S.A. et al., 2012). Using microwave and vinegar for 1 min increased Rg3 from undetectable to 1.13% in the Korean ginseng berry (Kim et al., 2013). In American ginseng, root (10.6 ± 0.4 mg/g) contains higher Rg3 than that from leaf (7.5 ± 0.9 mg/g) (Popovich and Kitts, 2004). In fact, Rg3 is mostly produced from PPD ginsenosides, such as Rb1, Rb2, Rc, and Rd by attacking the C-20 glycosidic bond through acid treatment or heat processing, and the optimum condition producing ginsenoside Rg3 from ginsenoside Rb1 is heat at 180°C for 30 min (Popovich and Kitts, 2004; Vo et al., 2015). Therefore, this heat-produced Rg3, the intermediate metabolite of red and black ginseng, contributes to the red/black ginseng efficient benefits including anti-cancer (Keum et al., 2000), vasculature-protective (Lee M.R. et al., 2011), and anti-obesity. Currently, commercial use of Rh2 and Rg3 are produced by chemical (mild acidic conditions) (Han et al., 1982) or biological deglycosylation (microorganisms or glycoside hydrolases) (Bae S.H. et al., 2014) of the major protopanaxadiol-type ginsenosides. BST204, a ginsenoside extract containing a high concentration of Rh2 and Rg3 mixtures, was prepared from crude ginseng with a ginsenoside-β-glucosidase combined with acid hydrolysis (Bae S.H. et al., 2014). Recently, yeast cell factories are created to produce Rh2 or Rg3 from simple sugars (Wang et al., 2015), which may be an alternative approach to replace the traditional method of extracting these rare ginsenosides Rg3 and Rh2 from *Panax* plants.

High hydrostatic pressure extract fresh ginseng and water extract of red ginseng inhibit fat accumulation in 3T3-L1 cells (Lee et al., 2015). Individual Rg3 (20–40 μM) has been reported in attenuating differentiation in 3T3-L1 cells companied by regulating AMPK and PPAR-γ signaling pathways (Hwang et al., 2009). Our results are in line with these studies that Rg3 dose-dependently (30–100 μM) inhibited 3T3-L1 cells differentiation, attenuated expressions of critical markers of PPAR-γ, C/EBP-α, FAS, and perilipin. Moreover, Rh2, one of the major metabolite in the blood circulation after oral intake of Rg3 in rats (Fan et al., 2016), dose-dependently (at 20 and 40 μM) decreased fat accumulation in 3T3-L1 cells (Hwang et al., 2007). Similarly, Rg5 and Rk1, degraded from Rg3 by dehydration at the C-20 position (Lee et al., 2009), also can reduce lipid accumulation in 3T3-L1 cells although they are less effective than Rg3 (Kim et al., 2009). In addition, Rg3 at concentrations of 1–10 μM significantly increased glucose uptake and expressions of glucose transporter 4 (GLUT4), insulin receptor substrate (IRS-1), and phosphatidylinositol 3-kinase (PI3K)-110α protein in 3T3-L1 cells (Lee O.H. et al., 2011). These results suggest that Rg3 and its metabolites Rh2, Rg5, and Rk1 may be potential agents in reducing adipogenesis.

3T3-L1 cells are widely used to study pre-adipocyte differentiation because this cell line provides a homogeneous population with virtually all cells being at the same stage. However, 3T3-L1 pre-adipocytes are embryonal in origin, are aneuploid and undergo early clonal expansion during differentiation in culture (Cornelius et al., 1994). HPAs, including both pre-adipocytes and fibroblast-like cells isolated from human adults, are diploid, and differentiate in culture without a clonal expansion phase (Entenmann and Hauner, 1996). These differences may contribute to the different sensitivity of compounds in inhibiting differentiation in our study that minimum 30 μM of Rg3 was required in 3T3-L1 cells, but only 10 μM of Rg3 can significantly reduced fat accumulation and relevant proteins in HPAs. This observation is in line with a recent study that combined genistein, quercetin, and resveratrol synergistically inhibited human pre-adipocytes differentiation at lower levels than that in 3T3-L1 cells. Moreover, 3T3-L1 cells and HPAs react different in the apoptosis regulation study (Papineau et al., 2003). In addition, this effective Rg3 concentration (10 μM) can be reached (10.2 μM) in 2 h after oral ginsenoside Rg3 (50 mg/kg) in normal rats (Fan et al., 2016), indicating that this Rg3 anti-obesity effect can apply to humans with Rg3 supplements. Thus, this lower concentration requirement in HPAs further supports our hypothesis that Rg3 might be an agent to prevent/reduce obesity in humans.

Red ginseng crude extract has been reported in preventing obesity in rodent models by reducing leptin level and adipogenesis level (Lee et al., 2010; Lee S.H. et al., 2012; Song et al., 2012) as well as enhancing fatty acid oxidation and energy expenditures via activation of PPAR-α in rats (200 mg/kg to 10 week-old, for 32 weeks) (Lee et al., 2007). However, there is no report that dietary individual Rg3 prevents obesity in animals. In the present study, we did not observe significantly changes of body weight, food intake, and fat pad by dietary intake of Rg3 (0.01%, w/w) in ob/ob mice. But we found that Rg3 significantly reduced PPAR-γ and C/EBP-α protein expressions in liver, fasting blood glucose and two major endogenous antioxidant molecules GS and GST, indicating that Rg3 has the potential to fight obese, at least in liver, the factory of energy metabolism and adipogenesis. The low dosage (0.01%, w/w) of Rg3 may contribute to the failure of weight gain and fat pad changes. Because of the complexity of the *in vivo* physiological environment, responses to treatments in pre-adipocytes *in vivo* may differ from those in 3T3-L1 and HPA cells. Although we are not sure, ob/ob mice with normal diet may be not as efficient as the high-fat-induced obese mice in this kind of studies. In addition, there is no clinical anti-obesity Rg3 report to our knowledge. In the future human anti-obesity studies, we recommend to apply Rg3 to persons at the first year of life and prepuberty, which is based on the fact that fat cells number increase is highest during these ages (van Harmelen et al., 2003) and the inhibitory effect in pre-adipocytes differentiation of Rg3 as we discussed in this study. All these issues should be further addressed in the future studies.

Taken together, we found that Rg3 dose-dependently inhibited cell differentiation, and expressions of adipogenic markers including PPAR-γ, C/EBP-α, FAS, and perilipin in 3T3-L1 cells. We are the first found that ginsenoside Rg3 inhibited pre-adipocyte differentiation in HPAs at much lower concentrations compared to the concentrations required in rodent pre-adipocytes 3T3-L1 cells. We are also the first reporter that dietary supplementation Rg3 attenuated hepatic protein expressions of PPAR-γ and C/EBP-α, key regulators of adipogenesis and fat metabolism, as well as the improvements in fasting blood glucose and hepatic antioxidants GR and GST in obese mice. These results suggest that ginsenoside Rg3 may be a potential agent in reducing/preventing obesity.

## Author Contributions

LyZ, LjZ, and XW conducted the experiments and prepared the draft. HS designed and directed the experiments, revised and improved the manuscript. All authors have read and agreed on the finally submitted version of the manuscript.

## Conflict of Interest Statement

The authors declare that the research was conducted in the absence of any commercial or financial relationships that could be construed as a potential conflict of interest.

## Abbreviations

C/EBP-α: CCAAT/enhancer binding protein alpha
FAS: fatty acid synthase
FABP4: fatty acid binding protein 4
GR: glutathione reductase
GST: glutathione *S*-transferase
HPAs: human primary adipocytes
IBMX: 3-isobutyl-1-methylxanthine
MDI medium: IBMX + dexamethasone + insulin medium
PBS: phosphate-buffered saline
PCR: polymerase chain reaction
PPAR-γ: peroxisome proliferator-activated receptor gamma.
